# Gene therapy with AAV2-CDNF provides functional benefits in a rat model of Parkinson's disease

**DOI:** 10.1002/brb3.117

**Published:** 2013-01-14

**Authors:** Susanne Bäck, Johan Peränen, Emilia Galli, Päivi Pulkkila, Liina Lonka-Nevalaita, Tuulia Tamminen, Merja H Voutilainen, Atso Raasmaja, Mart Saarma, Pekka T Männistö, Raimo K Tuominen

**Affiliations:** 1 Division of Pharmacology and Toxicology Faculty of Pharmacy, University of HelsinkiHelsinki, Finland; 2 Institute of Biotechnology, University of HelsinkiHelsinki, Finland; 3 Division of Biopharmaceutics and Pharmacokinetics Faculty of Pharmacy, University of HelsinkiHelsinki, Finland

**Keywords:** 6-OHDA, AAV, CDNF, GDNF, gene therapy

## Abstract

Cerebral dopamine neurotrophic factor (CDNF) protein has been shown to protect the nigrostriatal dopaminergic pathway when given as intrastriatal infusions in rat and mouse models of Parkinson's disease (PD). In this study, we assessed the neuroprotective effect of CDNF delivered with a recombinant adeno-associated viral (AAV) serotype 2 vector in a rat 6-hydroxydopamine (6-OHDA) model of PD. AAV2 vectors encoding CDNF, glial cell line–derived neurotrophic factor (GDNF), or green fluorescent protein were injected into the rat striatum. Protein expression analysis showed that our AAV2 vector efficiently delivered the neurotrophic factor genes into the brain and gave rise to a long-lasting expression of the proteins. Two weeks after AAV2 vector injection, 6-OHDA was injected into the rat striatum, creating a progressive degeneration of the nigrostriatal dopaminergic system. Treatment with AAV2-CDNF resulted in a marked decrease in amphetamine-induced ipsilateral rotations while it provided only partial protection of tyrosine hydroxylase (TH)-immunoreactive cells in the rat substantia nigra pars compacta and TH-reactive fibers in the striatum. Results from this study provide additional evidence that CDNF can be considered a potential treatment of Parkinson's disease.

## Introduction

Neurotrophic factors regulate plasticity, promote survival, and protect adult neurons from toxins and injury (reviewed by Petrova et al. 2004 and Lindholm and Saarma 2010). They are therefore considered potential drug candidates for the treatment of neurodegenerative diseases, such as Parkinson's disease (PD), where progressive loss of midbrain dopamine (DA) neurons affects motor performance (Dauer and Przedborski 2003).

Cerebral dopamine neurotrophic factor (CDNF) and mesencephalic astrocyte–derived neurotrophic factor (MANF) represent an evolutionary conserved family of neurotrophic factors (Lindholm et al. 2007; Parkash et al. 2009; Lindholm and Saarma 2010). In a rat 6-hydroxydopamine (6-OHDA) model of PD, single injections of CDNF and MANF proteins were able to restore function and increase survival of midbrain DA neurons (Lindholm et al. 2007; Voutilainen et al. 2009). In the same model, also a 2-week continuous infusion of CDNF attenuated the degeneration of the nigrostriatal DAergic system (Voutilainen et al. 2011). CDNF had also a significant neuroprotective and neurorestorative effect in the mouse 1-methyl-4-phenyl-1,2,3,6-tetrahydropyridine (MPTP) model of PD (Airavaara et al. 2012). In addition, MANF had survival-promoting effect on DA neurons in vitro (Petrova et al. 2003), and intracellularly delivered MANF was able to block Bax-induced neuronal apoptosis (Hellman et al. 2011). The invertebrate analog *Drosophila melanogaster* MANF (DmMANF) has also been proven to be crucial for the maturation of the nervous system and for the maintenance of DAergic neurons in *D. melanogaster* (Palgi et al. 2009). These observations point out a possible implication for CDNF/MANF family of neurotrophic factors in the treatment of PD.

Glial cell line–derived neurotrophic factor (GDNF) has been considered the most promising neurotrophic factor, showing positive effects in several animal models of PD (Hoffer et al. 1994; Kearns and Gash 1995; Tomac et al. 1995a; Gash et al. 1996; Zhang et al. 1997; Kirik et al. 2004), but not in the α-synuclein model of PD (Decressac et al. 2011). Controversial results from clinical trials with GDNF (Gill et al. 2003; Nutt et al. 2003; Slevin et al. 2005; Lang et al. 2006) have pointed out the importance of effective and reliable administration techniques (discussed by Sherer et al. 2006; Ramaswamy et al. 2009). Indeed, more emphasis should be paid on the delivery methods, as intracranial infusion has been associated with large variability in the diffusion of the protein (Sherer et al. 2006). Furthermore, following sustained intraputamenal delivery, neutralizing antibodies against GDNF could be detected in some patients, probably due to leakage of delivery system. Gene therapy using recombinant viral vectors may be one answer to these problems (reviewed by Björklund and Kordower 2010).

Adeno-associated virus (AAV) has a beneficial profile, with low toxicity, and allowing long-term gene expression (reviewed by McCown 2005). It has become the most common vector for gene transfer in clinical trials in PD patients (Kaplitt et al. 2007; Marks et al. 2008, 2010; LeWitt et al. 2011). The delivery of the GDNF family neurotrophic factor neurturin (NRTN) using an AAV2 vector was recently proven to be safe and well tolerated in PD patients, even though the clinical outcome was rather modest (Marks et al. 2008, 2010). One major concern when delivering therapeutic agents with viral vectors is that the level of the expression is difficult to control, and sustained expression of the transgene could cause negative effects. This problem could be solved in the future as more efficiently controlled inducible vectors are being developed.

On the basis of our previous results on the neuroprotective and neurorestorative effects of CDNF protein in PD rat and mouse models (Lindholm et al. 2007; Voutilainen et al. 2011; Airavaara et al. 2012), we studied the effect of CDNF gene delivery using an AAV serotype 2 vector encoding CDNF. The protein expression following the gene transfer was analyzed using specific enzyme-linked immunosorbent assay (ELISA) and the neuroprotective effect of the AAV2-CDNF gene therapy was compared with that of AAV2-GDNF (positive control) and AAV2-GFP (green fluorescent protein) and PBS (phosphate-buffered saline) (negative controls).

## Materials and Methods

### Construction, purification, and characterization of AAV2 vectors

**Figure 1:**
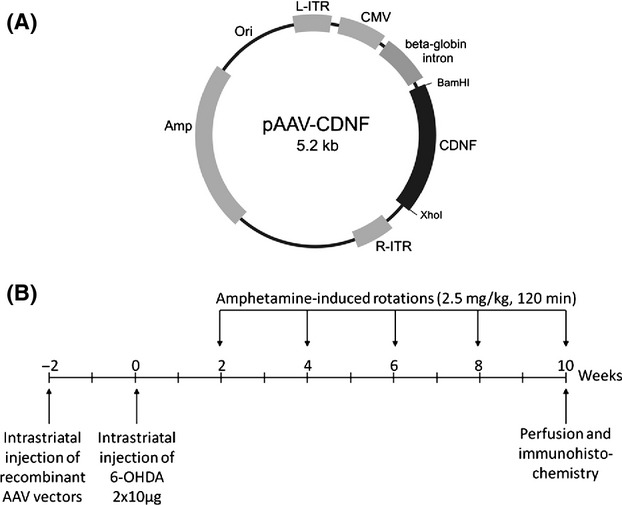
Schematic drawing of the pAAV2-CDNF vector (A) and experimental design for evaluating the neuroprotective effect of AAV2-CDNF in a 6-OHDA partial lesion model of PD in rats (B).

The open reading frame of human CDNF (hCDNF) was cloned into the *BamH*I/*Xho*I sites of pAAV-MCS (Stratagene, La Jolla, CA) to create pAAV-CDNF (Fig. 1A). The pAAV-CDNF, pAAV-GDNF (Lonka-Nevalaita et al. 2010), and pAAV-hrGFP (Stratagene) plasmids were cotransfected with the pHelper and the pAAV-RC (Stratagene) plasmids into AAV-293 cells by the CaCl_2_ method (National Virus Vector Laboratory, University of Eastern Finland, Kuopio, Finland) according to the manufacturer's instructions (Stratagene). About 48-h posttransfection, the cells were lysed by cycles of freeze/thaw, and the extracted recombinant AAV2 viruses were purified by centrifugation using a CsCl gradient. The virus sample was dialyzed in PBS containing 12.5 mmol/L MgCl_2_. The titer was determined by quantitative polymerase chain reaction (PCR) (SYBR Green technique with primers for the cytomegalovirus (CMV) promoter) for AAV2-CDNF (1 × 10^12^ virus genomes [vg]/mL), AAV2-GDNF (5.18 × 10^11^ vg/mL), and AAV2-GFP (4 × 10^10^ vg/mL). The in vitro transduction efficiency of AAV2-CDNF was determined by applying the virus particles to HeLa cells that were then stained by anti-CDNF antibody to verify CDNF expression. The expression of all recombinant proteins was driven by the CMV promoter.

### Animals and surgery

#### Animals

Wistar male rats (Harlan, the Netherlands) were group-housed under standard laboratory conditions in a 12 h/12 h dark/light cycle with free access to rodent food and fresh tap water. All animal procedures were reviewed and approved by the National Animal Experiment Board (ESLH-2009-05234 Ym-23) and carried out in accordance with the European Communities Council Directive 86/609/EEC.

#### AAV2 vector injection

Rats (250–300 g) were anesthetized with isoflurane (4% induction, 2.5–3.0% maintenance) and the recombinant AAV2 viral vectors were injected into the rat striatum in a stereotaxic operation. To target the striatum, viral vectors were given as single injections into the left hemisphere, 1.0 mm anterior and 2.7 mm lateral to bregma, and 5.0 mm below the dura (stereotaxic coordinates according to Paxinos and Watson 1997).

For behavioral experiments and analysis of cell survival after lesioning, rats were randomly divided into six treatment groups (*n* = 9–10/group) receiving three different doses of AAV2-CDNF (4.0 × 10^7^, 2.0 × 10^8^, 1.0 × 10^9^ vg/striatum), AAV2-GDNF (1.0 × 10^9^ vg/striatum), or one of the two negative controls (AAV2-GFP 2.0 × 10^8^ vg/striatum or PBS).

For analysis of protein expression, rats were injected with AAV2-CDNF 4.0 × 10^7^ (*n* = 4), 2.0 × 10^8^ (*n* = 4), or 1.0 × 10^9^ vg (*n* = 20), or AAV2-GDNF 1.0 × 10^9^ vg (*n* = 3) into the left striatum. The right striatum was left intact, or injected with AAV2-GFP or PBS. Injections were done using a stereotaxic injector (Stoelting, Wood Dale, IL) and 10-μL syringes (Hamilton, Bonaduz, Switzerland). Injection volume was set to 5 μL (AAV2 viral stocks were, if necessary, diluted with PBS) and injection speed was 1 μL/min, leaving the needle in place for 2 min before withdrawal. Rats received tramadol 1 mg/kg subcutaneously (s.c.) for postoperative pain and were kept in single cages overnight.

#### Lesions

For all rats in the neuroprotection study, lesioning of the midbrain DAergic system was done 2 weeks after viral vector injections using 6-OHDA (6-OHDA hydrochloride; Sigma, St. Louis, MO) (Fig. 1B). Thirty minutes before the 6-OHDA injections, rats received desipramine 15 mg/kg intraperitoneally (i.p.) (desipramine hydrochloride, Sigma) to protect noradrenergic nerve terminals from the toxin. 6-OHDA was injected under isoflurane anesthesia using stereotaxis as described above. Rat received two injections, each 10 μg of 6-OHDA (2.5 μg/μL, 1 μL/min), into the left striatum according to following coordinates: A/P +1.6, M/L +2.2, and A/P −0.4, M/L +4.0, 5.0 mm below dura.

### Amphetamine-induced rotational behavior

Behavioral tests were carried out 2, 4, 6, 8, and 10 weeks after 6-OHDA injections (Fig. 1B). Rats received an i.p. injection of 2.5 mg/kg d-amphetamine, and complete (360°) amphetamine-induced rotations were monitored for 120 min using Rotorat software (Med Associates, Inc., St. Albans, VT). Before injecting the animals with amphetamine, rats were attached to the equipment and spontaneous rotations were registered for 30 min. Results are given as net ipsilateral (ipsilateral minus contralateral) full turns. As there was no significant difference between the two control groups (vehicle and AAV2-GFP), results from these groups were pooled together to one control group (Kirik et al. 2000).

### Immunohistochemistry

Ten weeks post lesion (12 weeks after AAV2 injection), rats were perfused after receiving an overdose of pentobarbital (90 mg/kg, i.p.; Mebunat®, Orion Oyj, Espoo, Finland). Rat brains were fixed with 4% paraformaldehyde for 10 min by intracardial perfusion, removed, and postfixed for an additional 4 h floating in paraformaldehyde. After storage in sucrose, the brains were frozen and cut on a sliding microtome into 40-μm-thick sections in series of six.

Immunohistochemical staining was done on free-floating brain sections as described previously (Voutilainen et al. 2011). Primary antibodies used were mouse monoclonal anti-tyrosine hydroxylase (anti-TH, 1:2000, #MAB318; Millipore, Temecula, CA), rabbit polyclonal anti-CDNF (1:10 000, #4343; ProSci, Inc., Poway, CA), goat polyclonal anti-GDNF (1:3000, #AF-212-NA; R&D Systems, Minneapolis, MN), and secondary antibodies biotinylated horse-anti-mouse, goat-anti-rabbit, and horse-anti-goat (1:200; Vector labs, Burlingame, CA), respectively. Staining was reinforced using avidin–biotin–enzyme complex (ABC-kit; Vector labs) and visualized using diaminobenzidine (DAB) as a chromogen.

For colocalization studies, double immunofluorescence labeling was applied. After labeling of CDNF, brain sections were incubated in fluorescein-conjugated goat anti-rabbit (1:500; Thermo Scientific, Pierce Biotechnology, Rockford, IL) for 2 h in room temperature. The brain sections were blocked in 10% normal rabbit serum, and labeled with mouse monoclonal anti-NeuN (1:1000, #MAB377; Millipore, Temecula, CA, +4°C) or mouse monoclonal anti-TH (1:1000; Millipore, room temperature) overnight. Texas Red-conjugated rabbit anti-mouse (1:1000; Thermo Scientific, Pierce Biotechnology) was used as secondary antibody and was applied on the sections for 2 h in room temperature.

For imaging and morphometric analyses, all neuroanatomical areas were identified according to the nomenclature of the rat brain atlas of Paxinos and Watson (1997). Immunohistochemistry photomicrographs (representative pictures) were achieved with Optronics digital camera (Goleta, CA) connected to a microscope (Olympus BX51; Olympus Optical, Tokyo, Japan). If necessary, the digital pictures were corrected for brightness and contrast with Adobe Photoshop CS5.1 software (Version 12.1; Adobe Systems Incorporated, Mountain View, CA). The effect on sprouting of TH-immunoreactive fibers was studied following both gene therapy and long-term protein infusion of the neurotrophic factors. Behavioral and morphometric data from the protein infusion study have already been published (Voutilainen et al. 2011). Lesions were done in the same way as in this study, and CDNF or GDNF proteins were infused into the lesioned striatum for 2 weeks, starting 2 weeks post lesion. Brains were fixed and immunohistochemically stained for TH 14 weeks post lesion (Voutilainen et al. 2011).

### Morphometric analysis

For assessment of TH-reactive cells in the substantia nigra pars compacta (SNpc) and TH-reactive fibers in the striatum, the immunohistochemically stained brain sections were blinded to the researcher.

#### Optical density in the striatum

For measurement of optical density of TH-reactive fibers in the striatum, pictures of the immunohistochemically TH-stained striatal sections were acquired with a digital camera (Nikon Corporation, Tokyo, Japan) attached to a stereomicroscope. TH-reactive fiber density in the striatum was assessed by measuring the density along a line drawn across the dorsal part of the striatum using Image-Pro Plus software (Media Cybernetics, Bethesda, MD). All density values were corrected for the background density. Three coronal sections from the striatum of each rat brain were analyzed, and the results are given as percentage of the lesioned striatum as compared with the intact striatum.

#### Stereologic assessment of TH-reactive cells in SN

The number of TH-reactive cells in SNpc was estimated according to the optical fractionator method combined with the dissector principle with unbiased counting rules using the Stereo Investigator platform (MicroBrightField, Williston, VT) (Lindholm et al. 2007; Voutilainen et al. 2009). Cells in SNpc were counted bilaterally in six sections (40-μm sections, every sixth section) from each brain ranging from approximately 4.5 to 6.0 mm posterior to bregma (Paxinos and Watson 1997). Results are given as percentage of cells in the lesioned rat SNpc as compared with the intact SNpc.

As there were no differences between the negative control groups (vehicle and AAV2-GFP) in either the amount of TH-reactive cells in the SNpc or TH-reactive fiber density in the striatum, the results from these groups were pooled together to one control group.

### Biochemical analysis of protein expression

#### Viral transduction of cells and preparation of tissue samples

To analyze the time-dependent protein expression following gene transfer with AAV2 vector, rats were injected with AAV2-CDNF 1.0 × 10^9^ vg in the left striatum, leaving the right striatum intact, and decapitated 1, 2, 4, 8, or 12 weeks after injection (*n* = 4/time point). Another group of animals was used for assessing the virus vector titer-dependent protein expression. These animals received an injection of AAV2-CDNF 4.0 × 10^7^, 2.0 × 10^8^, or 1.0 × 10^9^ vg into their left striatum, while the right striatum was used as a control (intact, or injected with AAV2-GFP or with PBS). All rats used for the titer-dependent expression analysis were decapitated 4 weeks after AAV2 vector injection. In a pilot study, GDNF expression following AAV2-GDNF injection was determined 9 weeks after viral vector injection (*n* = 3).

After decapitation, the brains were removed and the SN (2-mm punch from 1-mm section) and the striatum (in total) were collected and frozen. Samples were homogenized in 150 μL of lysis buffer (137 mmol/L NaCl, 20 mmol/L Tris, pH 8.2, 1% NP40, 10% glycerol, 1 mmol/L phenylmethanesulfonylfluoride, 0.5 mmol/L NaVO_3_, and Complete Mini protease inhibitor cocktail [Roche, Mannheim, Germany]) using a sonicator (Rinco Ultrasonics, Romanshorn, Switzerland). The tissue samples were centrifuged at 15,300*g* for 20 min (4°C), 1 mol/L HCl was added to the supernatant (pH <2), and the samples were incubated 30 min on ice. The pH of the samples was neutralized (pH 7.6) using 1 mol/L NaOH, and the samples were stored in 80°C until analysis.

#### CDNF-ELISA

Total CDNF concentration in the rat brain samples was analyzed with an in-house-built double-antibody sandwich ELISA specific for hCDNF using standard procedures. A detailed protocol for the CDNF-ELISA will be published elsewhere (E. Galli, M. Ustav, P. Taba, A. Urtti, M. Yliperttula, P. Pulkkila, and M. Saarma, unpubl. ms.). Briefly, for antigen capture, a 96-well microtiter plate was coated with antibodies against CDNF. To reduce unspecific binding, the antibody-coated wells were incubated with 3% bovine serum albumin (BSA) in PBS. After washing, homogenized brain tissue samples (or recombinant hCDNF at eight different concentrations ranging from 0–1000 pg/mL for a standard curve) were applied on the wells and incubated overnight at +4°C. The homogenized SN samples were diluted 1:4 and analyzed as duplicate. In the case of striatal samples, the control-side samples were diluted 1:4 and analyzed as triplicate, whereas the left-side AAV2-CDNF-injected samples were diluted 1:20, or in the case of lower vector titers (4.0 × 10^7^ and 2.0 × 10^8^ vg), samples were diluted 1:4, and analyzed as triplicate. On the following day, the plate was washed and a detection antibody against CDNF was added to the wells and incubated 3 h at 37°C. The detection antibody was produced in a different animal species from the coating antibody used. Finally, the formed antibody-CDNF-antibody “sandwich” complexes in the wells were visualized with a horse-radish peroxidase (HRP)-conjugated secondary antibody and 3,3′,5,5′-tetramethylbenzidine (TMB) substrate according to the manufacturer's instructions (DuoSetELISA Development System, R&D Systems). The concentration of hCDNF (pg/mL) measured by ELISA was compared with the total protein concentration (mg/mL) of each brain sample determined by modified Lowry method, with bovine gamma globulin as the standard (DC Protein Assay, Bio-Rad Laboratories, Hercules, CA).

Sensitivity of the CDNF-ELISA to human recombinant CDNF (Icosagen, Tartu, Estonia) was determined by calculating the mean response of ten blank samples and evaluating the mean plus three standard deviations on the standard curve. Specificity of the ELISA was tested by measuring cross-reactivity to recombinant mouse CDNF (R&D Systems) and to human recombinant MANF (Icosagen).

#### GDNF-ELISA

The amount of GDNF protein in the brain samples was analyzed using the GDNF E_max_® ImmunoAssay System (Promega, Madison, WI) according to the instructions from the supplier. The concentration of GDNF was compared with the total amount of protein in the sample.

### Statistical analysis

All results are given as mean values with error bars showing the standard error of the mean (SEM). Statistical analyses were performed using PASW Statistics 18 (SPSS, Inc., Chicago, IL). For normally distributed data, differences between treatment groups were determined with one-way analysis of variance (ANOVA) followed by Tukey honestly significant difference (HSD) post hoc test. In cases when Levene's test for homogenicity gave statistical significance, Games–Howell post hoc test was applied. Differences between treatment groups were considered statistically significant if *P* < 0.05.

## Results

### Characterization of viral vector-mediated expression in intact animals

#### Sensitivity and specificity of the in-house-built CDNF-ELISA

Sensitivity of the CDNF-ELISA was determined to a minimum concentration of 10 pg/mL of recombinant human CDNF. In cross-reactivity tests, the assay recognized only 5% of mouse CDNF at the concentration 2 ng/mL. Human recombinant MANF gave no signal in the assay (highest tested concentration was 500 ng/mL). The ELISA readings of hCDNF in all the control rat brain samples (intact, AAV2-GFP- or vehicle-treated hemispheres) were under the detection limit of the assay.

#### Time- and titer-dependent protein expression following AAV-CDNF injection in vivo

**Figure 2:**
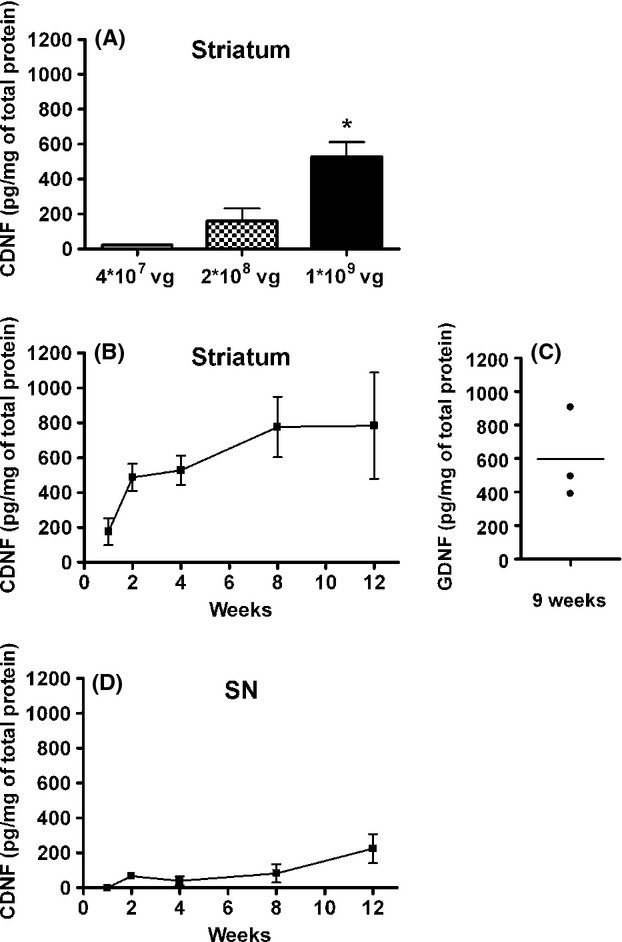
The level of hCDNF protein in the rat striatum (A, B) and substantia nigra (SN) (D) following injection of AAV2-CDNF into the striatum measured by our CDNF-ELISA assay (*n* = 4/measure point). The protein expression was dependent on the injected AAV2 vector titer, when measured 4 weeks after delivery of the hCDNF gene (A), remained stable until the end of the experiment (12 weeks) (B), and was comparable to that of GDNF following AAV-GDNF injection (C; *n* = 3). CDNF protein could also be detected in the ipsilateral SN following intrastriatal AAV2-CDNF injection (D). *P < 0.05 versus AAV-CDNF 4 × 10^7^ and 2 × 10^8^ vg, one-way ANOVA and Games–Howell post hoc test, *n* = 4/group (A, B, D), *n* = 3 (C).

The CDNF expression following intrastriatal AAV2-CDNF injection was monitored with CDNF-ELISA and showed both time and titer dependence (Fig. 2). A five-time increase in the injected virus vector titer (from 2 × 10^8^ to 1 × 10^9^ vg) resulted in a statistically significant increase from about 160 pg of CDNF/mg of total protein to about 530 pg of CDNF/mg of total protein (*P* < 0.05, one-way ANOVA [*F*_2,9_ = 16.792, *P* = 0.001] and Games–Howell post hoc test) (Fig. 2A). In the striatum (Fig. 2B), expression of hCDNF at about 180 pg/mg of total protein could be detected already 1-week postinjection (AAV2-CDNF 10^9^ vg), followed by a quite robust increase in the expression at 2-week postinjection (approximately 490 pg of CDNF/mg of total protein). The expression remained stable until the end of the study (12-week postinjection), when the total amount of hCDNF in the dissected striatum was 1.3 ± 0.5 ng (approximately 780 pg of CDNF/mg of total protein). After injecting AAV2-CDNF 10^9^ vg into the rat striatum, small amounts of hCDNF protein could also be detected in the SN starting at 2-week postinjection (Fig. 2D). In a small pilot study, the expression of GDNF 9 weeks after AAV2-GDNF injection was shown to correlate with the expression of CDNF (compare Fig. 2B and C).

#### Detection of protein expression with immunohistochemistry

**Figure 3:**
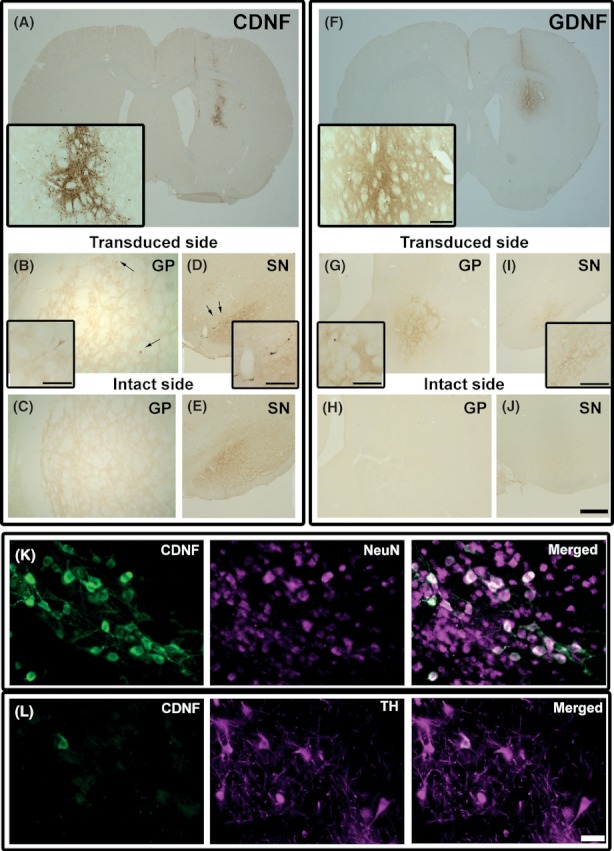
Expression of CDNF and GDNF proteins in the lesioned rat brain 12 weeks after injection of AAV2-CDNF or AAV2-GDNF into the striatum. Expression of CDNF (A) and GDNF (F) was detected around the injection tract and the expression of CDNF colocalized with the neuronal marker NeuN in the striatum (K). While CDNF protein was mainly found intracellularly, GDNF was evenly distributed in the tissue surrounding the injection tract (insets in A, F). Solitary CDNF-immunoreactive cells were detected in the ipsilateral globus pallidus (GP) (B) and substantia nigra (SN) (D). Some of the cells showing expression of hCDNF protein in the SNpc were TH positive (L). Following intrastriatal injection of AAV2-GDNF, GDNF signal could be detected in the GP (G) and in both SN pars compacta and SN pars reticulata (I). Background staining of CDNF and GDNF is shown in pictures C and E, and H and J, respectively. Scale bars: 200 μm (insets in A, F), 500 μm (B, C, D, E, G, H, I, J), and 10 μm (K, L).

Twelve weeks after intrastriatal injection of AAV2-CDNF, intensive CDNF signal was observed in the striatum around the injection tract (Fig. 3A). Compared with GDNF (Fig. 3F), the CDNF signal was to a larger extent found inside the transduced cells. CDNF-immunoreactive solitary cells were visible in the ipsilateral lateral globus pallidus (GP) and SNpc (Fig. 3B and D). The anti-CDNF antibody used recognizes also rat CDNF, and background staining from endogenous CDNF was observed in the contralateral GP and SN (Fig. 3C and E). Intrastriatal injection of AAV2-GDNF resulted in widespread GDNF immunoreactivity in the striatum, in the ipsilateral lateral GP, and in the SNpc and SN pars reticulata (SNpr) (Fig. 3F, G, and I). The majority of the CDNF-positive cells (green) in the striatum around the injection tract were NeuN positive (Fig. 3K), and the solitary CDNF-immunoreactive cells found in the SNpc showed colocalization with TH immunoreactivity (Fig. 3L).

### Amphetamine-induced rotations

**Figure 4:**
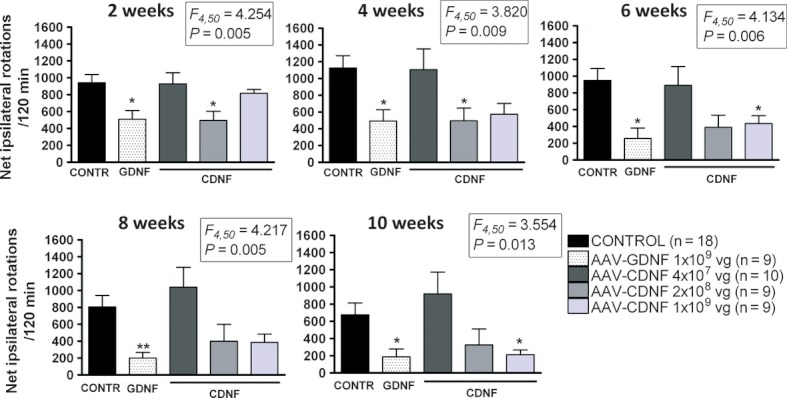
Amphetamine-induced (2.5 mg/kg, i.p.) rotational behavior was measured for 120 min at 2, 4, 6, 8, and 10 weeks following the unilateral 6-OHDA lesion. Intrastriatal injection of AAV2-CDNF or AAV2-GDNF was able to attenuate the ipsilateral turning behavior. Intergroup comparisons were assessed using one-way ANOVA (results showed in the upper right corner of each graph) and Games–Howell post hoc test. *P < 0.05, **P < 0.01 versus control group, *n* = 9-18.

At 2- and 4-week postlesion (4- and 6- week after viral vector injection), rats treated with AAV2-CDNF (2 × 10^8^ vg) showed a statistically significant reduction in amphetamine-induced (2.5 mg/kg, i.p.) net ipsilateral rotations as compared with the control group (results from one-way ANOVA and Games–Howell post hoc test in Fig. 4). At 6- and 10-week postlesion, AAV2-CDNF 1 × 10^9^ vg significantly improved the rotation asymmetry, while the lower titers (4 × 10^7^ and 2 × 10^8^ vg) had no effect. Treatment with AAV2-GDNF (1 × 10^9^ vg) showed therapeutic effect throughout the experiment (Fig. 4). Control rats showed a progressive increase in ipsilateral rotations until 6-week postlesion. After that, spontaneous recovery of the rotational behavior could be detected in the control groups.

### TH-immunohistochemistry

#### TH-reactive fiber density in the striatum

**Figure 5:**
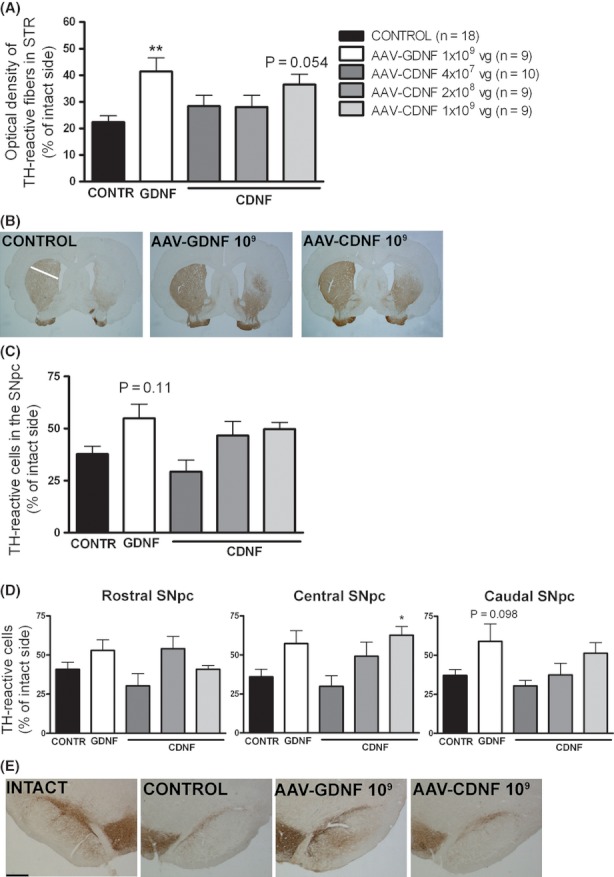
Tyrosine hydroxylase (TH) immunoreactivity in the rat striatum (A and B) and substantia nigra pars compacta (SNpc) (C, D, and E) 10 weeks post lesion (12 weeks after virus vector injection). Quantified results (A, C, and D) are given as percentage of the lesioned side as compared with the intact side. The white line across the dorsal striatum shows the site of optical density measurements (B). Treatment with AAV2-GDNF increased TH-reactive fiber density in the striatum as compared with the control group (A). Representative sections of TH staining in the striatum are shown in (B). Stereological analysis of nearly the entire SNpc (from 4.5 to 6.0 mm posterior to bregma) showed that none of the treatments were able to provide statistically significant protection of the TH-reactive neurons in the SNpc (C). AAV2-CDNF 1 × 10^9^ vg was able to protect the nigral TH-reactive cells in the central SNpc (D). Representative sections showing TH-reactive cells in the central SNpc are visualized in (E). *P < 0.05, **P < 0.01 versus control, one-way ANOVA, and Tukey HSD post hoc test, *n* = 9-18. Scale bar: 500 μm.

The amount of DAergic nerve terminals in the rat striatum 10-week postlesion was estimated by measuring the optical density of TH-reactive fibers. In control rats, there was an approximately 78% loss of TH-reactive fiber density as compared with the intact side (Fig. 5A and B). Treatment with AAV2-GDNF resulted in a statistically significant protection of the TH-reactive fibers compared with the control (58% loss of fiber density, *P* < 0.01, one-way ANOVA [*P* = 0.004, *F*_4,50_ = 4.350] and Tukey HSD post hoc test). In rats treated with AAV2-CDNF (10^9^ vg), an almost statistically significant increase in striatal TH-reactive fiber density was observed (63% loss of density, Tukey HSD post hoc test: *P* = 0.054).

#### TH-reactive cells in the SN

Ten weeks post lesion, TH-reactive cells in the SNpc were counted bilaterally in six sections, covering approximately 1400–1500 μm of the SNpc in the rostro–caudal direction. In the intact contralateral side, TH-reactive cell counts varied between 6500 and 11,600, with an average of 8650 ± 150 cells. There was no difference in the amount of TH-reactive cells in the intact side between the different treatment groups.

Ten weeks post lesion, an approximately 62% decrease in TH-reactive neurons could be detected in the lesioned SNpc in control rats (Fig. 5C). When taking into account all six nigral sections (ranging from approximately 4.5 to 6.0 mm posterior from bregma), none of the treatments resulted in significant protection of the TH-reactive cells. In rats treated with AAV2-GDNF, the cell loss was about 45% showing a trend toward protection of the TH-reactive cells (*P* = 0.11, one-way ANOVA [*P* = 0.012, *F*_4,50_ = 3.615] and Tukey HSD post hoc test).

When dividing the SNpc into a rostral, central, and caudal parts (two sections/part), we could conclude that the TH-reactive cell loss in the control group was consistent throughout all three areas (approximately 59%, 64%, and 63%, respectively). In the rostral part (ranging from about 4.5 to 5.0 mm posterior to bregma), no treatment effect on the TH-reactive cell counts could be seen (*P* = 0.065, *F*_4,50_ = 2.365, one-way ANOVA) (Fig. 5D). In the central part of the SNpc (ranging from about 5.0 to 5.5 mm posterior to bregma), treatment with AAV2-CDNF 1 × 10^9^ vg significantly protected the TH-reactive neurons (37% cell loss, *P* < 0.05, one-way ANOVA [*P* = 0.005, *F*_4,50_ = 4.193] and Tukey HSD post hoc test). Following treatment with AAV2-GDNF, the loss of TH-reactive cells was approximately 43%, but the result did not reach statistical significance (*P* = 0.139). In the caudal part (from about 5.5 to 6.0 mm posterior to bregma), treatment with AAV2-GDNF showed a trend toward protection of the TH-reactive neurons (41% cell loss as compared with 63% in the control group, *P* = 0.098, one-way ANOVA [*P* = 0.026, *F*_4,50_ = 3.033] and Tukey HSD post hoc test). In both the central and caudal parts, the rescue of TH-reactive neurons following AAV2-CDNF treatment showed titer dependence (Fig. 5D). While the same degree of protective effect of AAV2-GDNF treatment could be detected in all three subparts of the SNpc, the effect of AAV2-CDNF 1 × 10^9^ vg was mainly localized to the central parts of the SNpc.

#### Sprouting of TH-positive fibers

**Figure 6:**
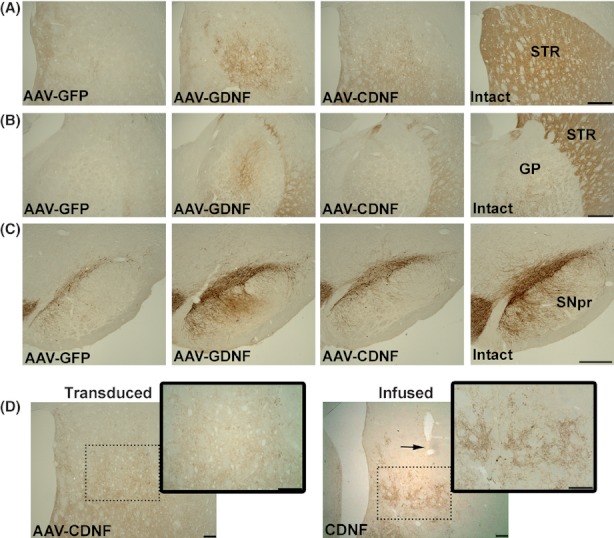
TH immunoreactivity in the striatum (STR) (A), globus pallidus (GP) (B), and substantia nigra pars reticulata (SNpr) (C) of the intact brain and 6-OHDA-lesioned rats treated with AAV2-GFP, AAV2-GDNF, or AAV2-CDNF. Twelve weeks post lesion, the 6-OHDA injections had caused an almost complete loss of TH-reactivity in the striatum, GP, and SNpr (AAV-GFP; A, B, and C). Injection of AAV2-GDNF 2 weeks before the 6-OHDA injections caused an increase of TH-positive fibers in the striatum, GP, and SNpr (AAV2-GDNF; A, B, and C). Treatment with AAV2-CDNF did not result in any clear sprouting, but caused an overall, partial protection of the TH-reactive fibers (A, B, and C) that was confirmed by the optical density measurements in the striatum (Fig. 5C). Compared with AAV2-CDNF, infusion of CDNF protein resulted in a denser meshwork of TH-reactive fibers close to the infusion tract (arrow) in the 6-OHDA-lesioned rats (D). Scale bars: 500 μm (A, B, C), 200 μm (D).

In rats treated with AAV2-GDNF prior to 6-OHDA lesioning, sprouting of TH-immunoreactive fibers in the striatum (Fig. 6A), lateral GP (Fig. 6B), and SNpr (Fig. 6C) could be seen. The areas that showed signs of sprouting corresponded to areas with GDNF immunoreactivity (compare Fig. 6B and C with Fig. 3E and F). Even if treatment with AAV2-CDNF showed a tendency to protect the TH-reactive fibers in the striatum (see above and Fig. 5C), no clear sprouting of TH-positive fibers in either of the studied brain areas was observed. When comparing 6-OHDA-lesioned rat brains treated with AAV2-CDNF or with CDNF protein, there were differences in the resulting pattern of TH-immunoreactive fibers in the striatum (Fig. 6D). Although AAV2-CDNF did not cause any clear sprouting in the striatum, treatment with CDNF protein (both 3 μg/24 h and 4.5 μg/24 h for 2 weeks) had an effect on striatal TH-positive fibers similar to that seen after AAV2-GDNF treatment (compare Fig. 6D and A, AAV2-GDNF) and treatment with GDNF protein (3 μg/24 h; picture not shown). Thus, the effect of intracellularly produced CDNF seems to differ from the effect of extracellularly applied CDNF.

## Discussion

The main result of this study is that intrastriatal CDNF gene therapy leads to expression of hCDNF in the brain and functional recovery of neural circuits controlling movements in 6-OHDA-lesioned rats. To the best of our knowledge, this is the first study of intracranial gene transfer of CDNF.

We have earlier shown that intrastriatal injection of CDNF protein either as a single dose (Lindholm et al. 2007) or as a 2-week continuous infusion (Voutilainen et al. 2011) is able to attenuate amphetamine-induced ipsilateral rotation asymmetry in unilaterally 6-OHDA-lesioned rats and protect TH-positive neurons in the SN against 6-OHDA toxicity. Recently, CDNF was shown to have neuroprotective and neurorestorative effects also in a mouse MPTP model (Airavaara et al. 2012). In this study, we were able to show that a single injection of AAV2-CDNF leads to prolonged expression of hCDNF in the brain. The protein expression was virus vector titer-dependent and hCDNF was measured from striatum 1 week after gene delivery and the expression remained stable up to at least 12 weeks. In the SN, expression of hCDNF was delayed and clearly seen only after 12 weeks. The amount of expressed CDNF and GDNF proteins was similar to each other at 8–9 weeks after virus vector injection. Thus, it is obvious that the neuroprotection observed in this study is due to expression of the neurotrophic factors in the brain.

The protection of TH-positive cells in the SN was rather modest. This may be related to the pattern of protein expression and the titers used. Thus, analyses of the striata revealed a much more spatially restricted expression of hCDNF as compared with that of GDNF. This may explain why the protection of TH-reactive cells in the SNpc in AAV2-CDNF-treated rats was seen mainly in the central subdivision of the SN. In AAV2-GDNF-treated rats, protection of TH-reactive cells seemed to be more consistent across all the analyzed nigral sections, although the effect was statistically nonsignificant. TH-reactive cells from different anterior–posterior levels of the rat SN have been reported to respond differently to both intrastriatal injections of 6-OHDA (Kirik et al. 1998) and to GDNF gene therapy (single intrastriatal injection of recombinant adenoviral vector) (Choi-Lundberg et al. 1998). Whether this is a consequence of the position of the striatal injection, spreading, and distribution of the neurotrophic factor or difference in the responsiveness of DAergic cells remains unclear. In a previous study, where approximately nine times bigger AAV2-GDNF titer dose was divided into three sites throughout the striatum intrastriatal AAV2-GDNF was shown to provide significant protection of nigral DAergic cells (Kirik et al. 2000). Therefore, it is likely that the titers used in this study were too low to provide maximal protection of the midbrain DAergic neurons against 6-OHDA toxicity. Also, the transduction volume of AAV serotype 2 is known to be low, and other serotypes providing better spread of the viral transgene and increased expression (e.g., AAV serotype 5; Burger et al. 2004) would probably also result in better protection of the nigrostriatal pathway. On the other hand, AAV2-NRTN (CERE-120) provided significant protection of nigral TH-reactive cells even at viral vector doses as low as 1.6 × 10^8^ vg (single-site injection of 6-OHDA) (Gasmi et al. 2007a).

Delivery of AAV2-GDNF prior to 6-OHDA administration provided an increase in TH-immunoreactive fiber density in the striatum, and sprouting of TH-immunoreactive fibers in the lateral GP and SNpr, as reported also by others (Kirik et al. 2000; Kordower et al. 2000; Georgievska et al. 2002a,b). We did not observe any sprouting of TH-positive fibers in the striatum of AAV2-CDNF-treated animals, even though the fiber density was partly preserved. The apparent difference between the two neurotrophic factors may be due to different patterns of expression of the proteins (see above). The immunostaining for GDNF was widespread while that of CDNF was almost entirely intracellular. Indeed, we noticed earlier that when CDNF was delivered as protein infusions into 6-OHDA-lesioned rat brain, it was able to induce signs of sprouting in the striatum (Voutilainen et al. 2011).

Both AAV2-CDNF and AAV2-GDNF reduced amphetamine-induced turning in a similar manner with maximal effect observed toward the end of the 12-week experiment. It is interesting to note that the effect of the lower titer of AAV2-CDNF (2 × 10^8^ vg) was statistically significant already at 2- and 4-week postlesion. At 6- and 10-week postlesion, only the higher titer (1 × 10^9^ vg) significantly improved the rotation asymmetry. The temporal differences in the statistical significances may reflect the rather big variation in response to amphetamine between individual animals. Starting from 6-week postlesion, a decrease in rotation asymmetry could also be seen in the control groups, masking to a minor extent the effect of the treatments. This kind of spontaneous recovery has been observed before, and it may be due to an increase of diffusion ability of DA in the striatum of lesioned rats, or an increase in the amount of DA released from remaining terminals (Doucet et al. 1986; Robinson et al. 1994) and/or regrowth of DAergic fibers (Blanchard et al. 1996). Even if the spontaneous recovery was taken into account, the higher titer of CDNF (1 × 10^9^ vg) had a significant behavioral effect in the turning model.

Our CDNF-ELISA was sensitive and specific for hCDNF, and confirmed the immunohistochemical findings. Thus, although most of the hCDNF protein was observed in the striatum, detectable amounts were also found in the SN. In our previous work, following a single intrastriatal injection, ^125^I-CDNF protein was transported to the SN and this transport could be blocked by unlabeled CDNF protein (Voutilainen et al. 2011). However, it is also possible that the AAV2-CDNF vector itself is transported from the striatum to SN (Paterna et al. 2004), inducing CDNF expression in the SN. In the medial part of SNpc, CDNF staining was colocalized with TH (a DA cell marker) which would support retrograde transport (of the protein or viral vector) along the DAergic nigrostriatal axis. Alternatively, CDNF protein in the SN could also be a result of anterograde transport along direct GABAergic projections (Fallon and Loughlin 1995). The CDNF immunoreactivity in some solitary cells in the ipsilateral lateral GP would also indicate anterograde transport (of protein or viral vector) via the indirect GABAergic pathway. GDNF is known to be transported in both a retrograde (through DAergic projections) and anterograde (through both direct and indirect GABAergic projections) fashion to the SN after intrastriatal injection of either the protein (Tomac et al. 1995b; Ai et al. 2003) or viral vectors encoding GDNF (Kirik et al. 2000; Kordower et al. 2000; Eslamboli et al. 2003; Johnston et al. 2009; Ciesielska et al. 2011). Also in our hands, AAV-GDNF-treated rat brain showed clear GDNF staining in the injected area (striatum), lateral GP, SNpc, and SNpr.

The site of delivery is one major open question regarding future gene therapy with neurotrophic factors in PD. When the nigrostriatal DAergic projections are lost, intraputamenal delivery of therapeutic agents to target the SN will probably require alternative routes of transportation. In this regard, GABAergic projections may play a significant role (Kirik et al. 2000; Ciesielska et al. 2011). It is also noteworthy that efficacy in a rodent model may not guarantee efficacy in humans. In animal models of PD, intrastriatal infusion of a recombinant AAV2-NRTN vector (CERE-120) was effective in behavioral tests, and NRTN immunoreactivity was traced to the striatum, GP, endopeduncular nucleus, SNpc, and SNpr (Kordower et al. 2006; Gasmi et al. 2007a,b; Herzog et al. 2007), suggesting both retrograde and anterograde transport. However, when CERE-120 was delivered into the putamen of PD patients, NRTN immunoreactivity was mainly restricted to the injected area, with a very scanty NRTN signal in the SN of postmortem brains (Bartus et al. 2011). The limited distribution of NRTN protein in the human brain may explain the very modest improvement in motor scores in the Phase 2 CERE-120 clinical trial (Marks et al. 2010). It is also consistent with the observation that in postmortem brains, there was very little induction of TH following intraputamenal infusion of CERE-120 (Bartus et al. 2011). In our study, only a scanty CDNF immunoreactivity could be detected along the anterograde indirect projections from the striatum to SN, and therefore, CDNF evidently would need the direct pathways. It is tempting to speculate that for an optimal clinical effect, AAV2-CDNF should be administered close to the SN, or to both the striatum and SN. Only when the CDNF receptor is identified and its location is found, further conclusions of the optimal administration site of CDNF can be made.

In conclusion, AAV2-CDNF was able to induce functional recovery of the rat midbrain neural circuitry to the same extent as AAV2-GDNF. AAV2-CDNF did not produce significant sprouting of neither TH-reactive fibers in the striatum nor increase in TH-positive cells in the SNpc. The modest neuroprotection is most probably due to rather low viral vector titers for both AAV2-CDNF and AAV2-GDNF and in the case of AAV2-CDNF, restricted and mainly intracellular expression of hCDNF protein. However, our results indicate that AAV2-CDNF is an alternative method for sustained delivery of CDNF protein in the brain. In the future, it would be important to find out the level of protection using higher titers, multiple injection sites, other vector serotypes, different promoter, and/or different injection site. In addition, more knowledge about the unique actions of CDNF on the midbrain DAergic transmission or other neuronal pathways are needed to assess the full potential of AAV2-CDNF as a therapy in PD.
